# Spontaneous neck hematoma associated with parathyroid adenoma

**DOI:** 10.1002/ccr3.9383

**Published:** 2024-08-28

**Authors:** Mitsumasa Okano, Mitsuko Yui, Masanori Teshima, Kazuhiko Sakaguchi

**Affiliations:** ^1^ Division of General Internal Medicine, Department of Internal Medicine Kobe University Graduate School of Medicine Kobe Japan; ^2^ Department of Otolaryngology‐Head and Neck Surgery Kobe University Graduate School of Medicine Kobe Japan

**Keywords:** acute neck pain, neck hematoma, parathyroid adenoma, parathyroid bleeding

## Abstract

We should consider parathyroid extraglandular bleeding for patients with acute neck pain and swelling. Evaluation of serum calcium and parathyroid hormone levels is crucial for a suspected neck hematoma associated with parathyroid adenoma.

## 1 CASE

**FIGURE 1:**
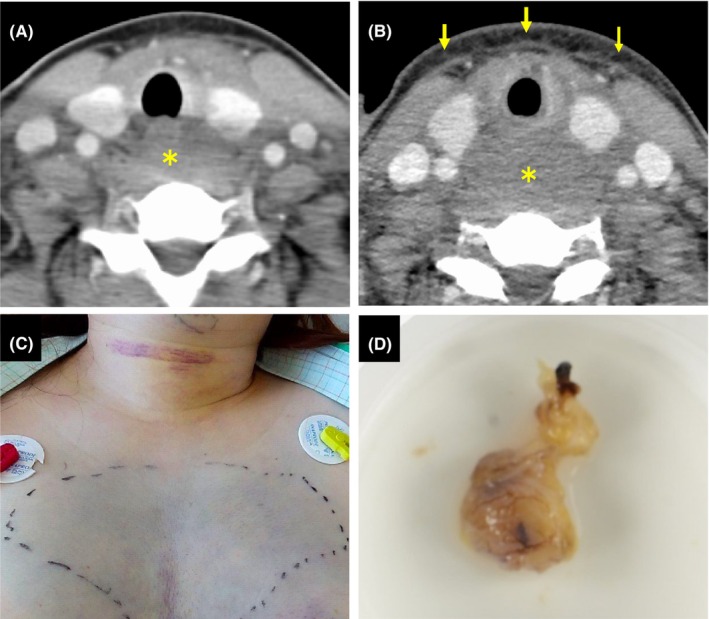
(A) Initial CT scan showed low‐density lesion behind the oropharynx without contrast enhancement. (B) Follow‐up CT scan showed a rapid increase in the size of the low‐density lesion (asterisk) with increased subcutaneous fat density (arrows). (C) Ecchymosis of the anterior neck and chest appeared. (D) Macro specimen of enlarged left parathyroid adenoma (7 × 6 × 4 mm).

A healthy 43‐year‐old woman presented with acute odynophagia and dysphagia, accompanied by anterior neck swelling. She had no history of trauma or medical procedures. She had no systemic symptoms of inflammation or infection. Physical examination revealed diffuse non‐tender swelling of the neck without rash. An initial CT scan revealed a low‐density lesion behind the oropharynx without contrast enhancement (Figure 1A). A follow‐up CT scan after 2 days demonstrated a rapid increase in the size of the lesion (Figure 1B, asterisk) with increased subcutaneous fat density (Figure 1B, arrows). In addition, ecchymosis of the anterior neck and chest appeared (Figure 1C). Laryngoscopy findings indicated a submucosal hematoma. Laboratory findings showed normal serum calcium levels and elevated PTH levels of 119 pg/mL. The level of soluble interleukin‐2 receptor, thyroid function, and coagulation function was normal. All the above findings, she was suspected of a hematoma associated with parathyroid adenoma. For a definitive diagnosis, we planned to perform a biopsy of the cervical swelling site under tracheal intubation. An incision was made in the posterior wall of the oropharynx using a mouth retractor, and a biopsy and culture test were performed. Based on these results, she was diagnosed with a hematoma, ruling out a tumor and abscess. The next day, surgery was performed through a skin incision on the anterior neck. An enlarged parathyroid gland and a blood clot located on the dorsal surface of the left thyroid lobe were removed (Figure 1D). Additionally, a hematoma in the anterior vertebral region was also removed. Histopathological assessment was consistent with parathyroid adenoma with hemorrhage. The PTH level was normalized within 3 days after surgery. She was discharged after tracheostomy decannulation.

## 2 DISCUSSION

Spontaneous neck hemorrhage is a rare and severe surgical emergency due to the traumatic rupture of vessels or extraglandular bleeding of the thyroid or parathyroid. A parathyroid extracapsular hemorrhage is a complication of parathyroid gland enlargement associated with hyperplasia, adenoma, and cancer. The precise mechanisms of such non‐traumatic bleeding are not known. Some previous reports suggested that blood supplies may occasionally fail to meet the increased demands caused by the lesions.^1^ This case highlights the importance of a diagnostic approach for patients who present with acute neck pain, swelling, and ecchymosis. In addition to a CT scan, clues to the diagnosis include a thorough endocrine history and elevated calcium and PTH levels.^2^, ^3^

## AUTHOR CONTRIBUTIONS

**Mitsumasa Okano:** Writing – original draft. **Mitsuko Yui:** Writing – review and editing. **Masanori Teshima:** Writing – review and editing. **Kazuhiko Sakaguchi:** Writing – review and editing.

## CONFLICT OF INTEREST STATEMENT

The authors have no conflict of interest to declare.

## CONSENT

Written informed consent was obtained from the patient to publish this report in accordance with the journal's patient consent policy.

## Data Availability

The data that support the findings of this study are available from the corresponding author upon reasonable request.
